# Strategies for recruitment and retention of diverse and underserved cancer survivor and caregiver dyads in clinical trials

**DOI:** 10.1016/j.conctc.2024.101425

**Published:** 2025-01-07

**Authors:** Mary Hadeed, Terry A. Badger, Chris Segrin, Rogelio Robles-Morales, Samantha J. Werts-Pelter

**Affiliations:** a Nursing and Health Science Division, University of Arizona College of Nursing, Tucson, AZ, USA; b University of Arizona Mel and Enid Zuckerman College of Public Health, Tucson, AZ, USA; c Department of Clinical Translational Sciences, University of Arizona College of Health Sciences, Tucson, AZ, USA; d University of Arizona Cancer Center, Tucson, AZ, USA; e Department of Communications, University of Arizona, Tucson, AZ, USA

**Keywords:** Recruitment and retention, Survivor-caregiver dyads, Cancer survivors, Diverse caregivers, Clinical trials, Rural, Underserved

## Abstract

**Background:**

Cancer survivor-caregiver dyads from underrepresented racial and ethnic groups and those with lower socioeconomic status are less likely to participate in clinical research. Sociocultural and socioeconomic barriers perpetuate health inequity and increase disparities in cancer care.

**Purpose:**

We describe our systematic approach to recruiting and retaining diverse survivor-caregiver dyads in supportive cancer care studies.

**Methods:**

Matsuda's research recruitment guidelines of evaluate, engage, reflect, and carefully match (“EERC”) were adapted and applied through a framework of six guiding principles.

**Results:**

A systematic approach to recruitment of underrepresented dyads in cancer support research includes 1) Developing a bilingual, bicultural study team with shared language and culture of the study population, 2) Ensuring team members share a passion for cancer health equity and are trained with a community-centric approach, 3) Designing accessible interventions, study materials, and shared data collection tools across similar studies with community and stakeholder input, 4) Engaging local and regional stakeholders with expertise of health disparities among the catchment area, 5) Partnering with Community Health Workers (CHWs) and gatekeepers to enhance community presence, and 6) Ensuring careful application of matching study team members and participants beyond race and ethnicity to prioritize the cultural values and social factors that impact cancer survivors and caregivers.

**Conclusion:**

Applying a systematic approach to recruiting and retaining underrepresented dyads in cancer research can potentially reduce sociocultural and socioeconomic barriers to cancer health equity.

## 1 Introduction

Diverse and underserved cancer survivors and caregivers are historically underrepresented in clinical research. Despite the National Institutes of Health (NIH) mandate to include minorities in federally funded research, clinical trials continually lack diverse enrollment [1]. Demographic representation of minorities in cancer clinical trials in the United States (U.S.) remains inadequate compared to their U.S. Census population parameters [2]. Clinical trials are critical to developing novel treatments and therapeutics, providing data crucial for Federal Drug Administration (FDA) decisions or other global agencies. As the “gold standard” in research design, randomized controlled trials (RCT) offer rigorous and reliable evidence for the relationship between exposure or intervention and outcomes. However, trials that are homogeneous in race and ethnicity lead to biased medical evidence [3], limit generalizability and may translate poorly in clinical settings.

Insufficient enrollment of diverse and underserved populations in cancer clinical trials contributes to health inequity and disparities in care. These inequities stem from systematic bias and unjust socioeconomic conditions. The historical underrepresentation of diverse, rural, or low socioeconomic status (SES) populations in research has led to disparate health outcomes among groups [2,3]. A systematic review of phase one oncology trials published between January and December 2019, found that patient enrollment did not reflect global or U.S. population demographics [3]. Non-Hispanic White representation heavily skewed enrollment in U.S. trials and globally. In cancer clinical trials, Asians and Latinos/as have the most pronounced underrepresentation, with Latino/a median representation of less than half of their U.S. population proportion [3]. Additionally, data indicate that Black Americans accounted for only 8.5 % of participants in oncology trials between 2010 and 2021, despite comprising nearly 14 % of the U.S. population [4]. This lack of diversity in cancer RCTs emphasizes the necessity for innovative recruitment and retention strategies.

### 1.1 Cancer among minorities and underserved populations in the United States

Less than 5 % of adults with cancer participate in trials [5], and only one in three clinical trial participants is a rural dweller [6]. In the Southwest U.S., the proportion of rural participation is even less [6]. Social determinants of health (SDOH), including SES, education, access to care, geographic location, and cultural values, influence cancer health outcomes and clinical trial participation [[7], [8], [9]]. Among diverse and rural populations, poorer cancer health outcomes arise due to informational, structural, and financial barriers that prevent timely, effective care [7,8]. For example, rural-dwelling Americans face sociodemographic barriers that influence high rates of cancer diagnoses and mortality, including lower educational attainment, high poverty rates, and limited access to health care [7,8,10]. Rurality contributes to geographical barriers to care, lifestyle, and environmental risk factors. Approximately 74 % of the U.S. landmass is considered rural, and 15 % of the U.S. population lives in a rural area [10], magnifying the divide in rural-urban cancer disparities. Rural residents are more likely to engage in risky health behaviors, tend to be older, and have less access to preventative care than their urban counterparts [10]. Socioeconomic barriers, such as low SES, significantly reduce participation in cancer clinical trials [5,11]. Recent studies have substantiated financial disparities in RCT enrollment. Unger and colleagues found patients with annual incomes less than $50,000 were 29 % less likely to participate in trials than patients with higher annual incomes [11]. Evidence suggests diverse and underserved survivor and caregiver dyads with low SES compounded by multiple SDOH factors are even less likely to be included in clinical trials [9].

The National Cancer Institute (NCI) has called for sustainable cancer control programs and dedicated research initiatives to address cancer disparities among rural and diverse communities [12]. Recently, NCI has funded investigators with supplements to strengthen cancer research efforts in rural and remote areas. Historically, NCI allocated only 3 % of its funding portfolio to research focused on underserved populations, with less than 1 % dedicated to racial and ethnic minorities [12,13]. The largest Phase 1 cancer trial cohort revealed a major urban-rural disparity, with less than 1.3 % of participants identifying as Hispanic or Latino and only 0.76 % as Hispanic or Latino and rural [14]. Moreover, diverse and rural regions, such as communities along the U.S.-Mexico border, are less likely to be included in research due to cultural differences, health literacy and language barriers, fear, and the perceived burden of clinical trials [[15], [16], [17]].

The sociopolitical climate among border regions contributes to misunderstandings of clinical research [16], including fears related to immigration status and lack of trust in researchers. Historical mistrust of U.S. healthcare delivery and research among racial and ethnic minorities is well documented [[1], [2], [3],[15], [16], [17]]. For Mexican-origin individuals living near the border, fear of deportation can impede access to health care, consequently preventing opportunities for research participation. Communities with concentrated immigration enforcement often experience racial profiling and immigration-related mistreatment, fostering a climate of stress and trauma for residents [18]. Rural and underserved communities experiencing high levels of migration have elevated mental and physical stress burdens [19]. Historic social, political, and cultural inequities have created deeply rooted structural health disparities among these communities. Insufficient national funding and sociodemographic and political barriers to participation underscore the misrepresentation of diverse groups in research. Accordingly, recruiting diverse and underserved populations into cancer research is critical to reducing health disparities among diverse cancer survivors and caregivers.

### 1.2 Caregivers as part of the healthcare team

Cancer caregivers, often unpaid family and friends who provide over half the survivor's care, are critical to the health care team, yet are underrepresented in research [20,21]. Caregivers offer both physical care and emotional support from diagnosis through survivorship. With the aging population, at-home health care is expanding, leading to more informal caregivers. Some researchers find engaging survivor-caregiver pairs in RCTs challenging, especially those from diverse groups. Recruitment challenges can arise from a lack of knowledge about the benefits of supportive care and symptom management interventions [22] or the lack of cultural adaptation of interventions for both survivors and caregivers [23]. Other factors, such as narrow caregiver inclusion criteria (e.g., intimate partners) or recruitment practices that conflict with cultural norms, further complicate engaging dyads. Badger et al. found that allowing the survivor to select the caregiver, including friends and family, was more culturally appropriate for the diverse community they were trying to reach [23]. Furthermore, a highly accessible, low-cost telephone-delivered intervention emerged as an effective way to retain diverse dyads in the research [23,24].

Caregivers and survivors have an interdependent relationship, and higher symptom burden and psychological distress in survivors often lead to increased stress for caregivers [[25], [26], [27]]. Caregivers frequently juggle roles by providing support during medical visits and managing information to protect the survivor's psychological health [28]. Protective buffering, although well-intentioned, can result in adverse psychosocial outcomes for either dyad member [29]. Bilingual caregivers, especially in Spanish-speaking communities, provide crucial informational support to linguistically diverse survivors, acting as language brokers to overcome patient-provider communication barriers. This role not only enhances health literacy but also reduces survivor depression rates [24,30], as cultural values like *confianza* (trust, confidence) and *respeto* (respect) empower Hispanic survivors to turn to trusted family members and friends for help, care, and support [31].

Sociocultural norms shape the unique caretaking roles diverse caregivers experience. Cultural influences impact caregiving outcomes such as self-efficacy and psychological distress [23,28,32]. Research suggests that African American caregivers may have a more positive view of caretaking and family obligation than non-Hispanic Whites because of their cultural values of familism [33]. Asian American and Hispanic caregivers report the highest levels of familial obligation, potentially increasing their emotional distress [34]. Within Hispanic families, the concept of *familismo* emphasizes shared medical decision-making, acting as both a protective factor and a source of stress by prioritizing a survivor's needs over the caregiver's [23,31,32]. Latina caregivers are especially at risk for mental distress due to feelings of domestic obligation, gender norms, and susceptibility to “role engulfment,” where caregiving defines their identity [35,36]. *Marianismo*, a cultural value marked by exceptional caregiving at the sacrifice of personal needs, contributes to caregiver stress [23].

Similar caregiving behaviors are notable in other racially and ethnically diverse populations. A recent national study found that Black and Hispanic caregivers provide more hours of care and complete more tasks than non-Hispanic Whites [20]. Cultural values among diverse populations may explain caregiving time and dedication differences. However, SES and financial factors also impact caregiving and quality of life. For example, a meta-analysis found ethnic minority caregivers had lower SES, worse physical health, and provided more care than White caregivers [33]. In another study, Fenton et al. found that Black and Hispanic caregivers reported more significant financial burdens than non-Hispanic Whites [37]. Socioeconomic factors propel an unequal distribution of resources among diverse and underserved populations, leading to more substantial financial burdens than their non-Hispanic White or urban counterparts. Health insurance is a major cost-contributing SDOH in cancer care. Lack of health insurance or public health coverage (e.g., Medicare and Medicaid) puts cancer survivors at high risk of financial toxicity [38]. Family caregivers of survivors with little to no insurance likely take on extra caregiving to meet the patient's care needs that would otherwise be covered by private insurance.

This paper reports on principles and strategies for recruiting and retaining diverse and underserved cancer survivors and caregivers in clinical research using exemplars from the Symptoms, Health, Innovation, and Equity (SHINE) Cancer Research group. Several SHINE cancer trials have recruited over 40 % Hispanic or Latino/a participation [39,40], with two exclusively involving rural Hispanic or Latina breast cancer survivors and caregivers [24,41]. Our methodology operationalizes Matsuda's Evaluate, Engage, Reflect, and Carefully Match (EERC) framework [42], emphasizing dyadic survivor-caregiver engagement in supportive care trials. The principles contribute to the existing literature by focusing on multilevel (e.g., interpersonal, social network, community) interventions for cancer survivors and caregivers.

## 2 Methods

We developed and refined strategies utilizing Matsuda's EERC framework to engage diverse, underserved populations in clinical trials, refining our approach over twenty years. This iterative process highlighted the EERC framework as an effective model for adaptation. The framework has been successful in other trials recruiting diverse and underserved populations [43,44]. The six recruitment guiding principles are based on studies focused on symptom management interventions over two decades. Across the exemplars, we have successfully enrolled hundreds (>700) of ethnically and racially diverse survivors [39], and survivor-caregiver dyads [24,40,41]. in urban and rural areas, and more than half consistently report incomes below $40,000 [[39], [40], [41]]. The following sections identify the approaches that helped shape the recruitment guiding principles.

### 2.1 Development of the guiding principles using Matsuda's EERC framework

**Fig. 1:**
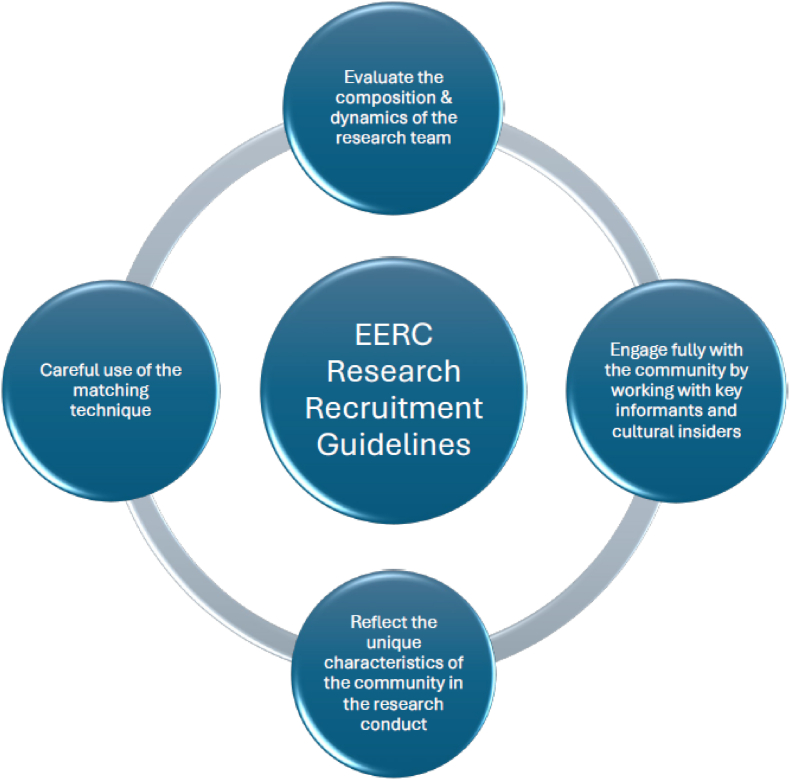
Matsuda's research recruitment guidelines: EERC

We identified six guiding principles for a systematic approach to engaging diverse dyads in research. EERC consists of the following components (Fig. 1): **e**valuate the composition and dynamics of the research team; **e**ngage fully with the community by working with key informants and cultural insiders; **r**eflect the unique cultural characteristics of the community in the research conduct; and **c**arefully use a matching technique [42]. The six principles (Table 1): 1) Team Development, 2) Shared Passion, 3) Collaboration, 4) Community Engagement, 5) Partnership, and 6) Careful Matching, operationalize EERC by emphasizing researcher reflexivity, cultural competence, and community engagement to enhance health equity and retain diverse participants.

**Table 1:**
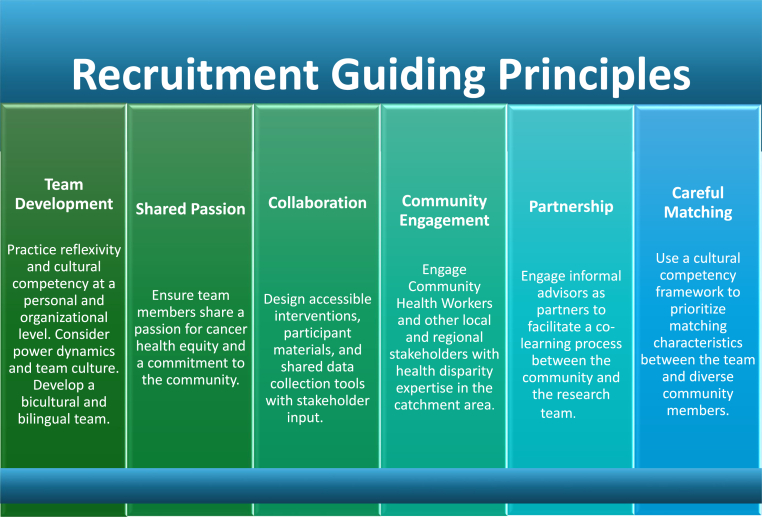
Recruitment guiding Principles.

## 3 Results

### 3.1 Team Development

Evaluating the research team through a team science approach is crucial, as it considers personal characteristics and situational factors that structure team dynamics and impact the science [42,45]. Team development should be considered at the project's inception to foster cohesiveness and connection. Strong research teams include individuals with diverse academic and professional experience, cultural upbringing, and communication styles. During team formation, attention to team dynamics and power differentials in different contexts is critical to team culture [42,46].

Extending team evaluation to include cultural competence and reflexivity is essential. Cultural competence, an evidence-based approach to healthcare delivery and community engagement, requires the team to consider policies, practices, behaviors, structures, and attitudes to work effectively with diverse cultures [47]. This framework applies at both personal and organizational levels. Researchers must self-assess their cultural knowledge and integrate culture and language into the research process by connecting with diverse community members to fill gaps in the researcher's background. A culturally dynamic, bilingual team adds value to the research.

#### 3.1.1 Developing a diverse team

Our multidisciplinary team included researchers from Universities and a Federally Qualified Health Center (FQHC). Fields of expertise included nursing, public health, biostatistics, social sciences, medicine, and cultural experts from the community. Investigators conceptualized the team at the start of each project to ensure they carried out each research stage effectively. Understanding the barriers to engaging diverse populations in research, we prioritized the following key practices in developing a diverse team.

More than half of the research staff members were bilingual and bicultural, with over twenty years of combined community outreach experience. All spoke Spanish and English and took a Spanish language proficiency exam administered by the ALTA Language Services [48], which included a live conversational exam on a medical subject. The team understood that building a linguistically diverse team was crucial to engaging diverse survivors and caregivers in our catchment area.

Team members were cross-trained in various tasks and encouraged with opportunities to gain experience across projects. For example, a staff recruiter cross-trained to learn project management. This approach allowed investigators to leverage skilled staff for various research roles and maintain the core team. Team members trained each other, and cross-training addressed power imbalance by providing a fair distribution of duties and encouraging collaboration across studies. Further, team members recruited and selected new members when needed, using their knowledge of the community and relationships with community members.

To foster collaboration and shared decision-making, the team held weekly in-person meetings with a Zoom option for those offsite. These meetings offered a comfortable space to discuss issues, share innovative ideas, and receive team feedback. The iterative nature of the meetings led to the development of best practices for engaging diverse survivors and caregivers.

### 3.2 Shared Passion

A team with a shared passion for connecting survivors and caregivers with resources, education, and clinical trial access promotes study success. This shared passion also extends to community commitment. Working with people who share these experiences enhances community-focused research and fosters high-level engagement [49].

#### 3.2.1 Sharing a passion for the research

Although individual areas of expertise varied, team members shared a passion for health equity in cancer care. During budget planning, we prioritized travel for community engagement. In addition to investigator travel, research staff attended 3 or 4 out-of-town health equity conferences or community events annually. Additionally, staff attended local community events at least 1 to 2 times monthly. Recruiters engaged with at least 25 interested community members per event, typically recruiting 2–4 participants. Events allowed research staff to engage providers, often at the request of the Community Advisory Board (CAB), made up of cancer survivors, academicians, and CHWs who reside in the community where the research is conducted. For instance, attending the Arizona Community Health Workers Association annual meeting facilitated interaction with some of the region's 2000 CHWs and Promotoras [50]. Regularly connecting with local cancer survivors, caregivers, and community providers preserved the team's strong enthusiasm and kept health equity at the forefront of the research.

### 3.3 Collaboration

Research designs that leverage existing community resources and focus on community collaboration increase clinical trial access for underserved populations [51]. Successful community collaboration relies on strong team-based efforts and consistent communication across multiple studies. Studies with overlapping inclusion criteria must coordinate before approaching a community to avoid overburdening and conflicting priorities. This approach can include using aligned data collection tools, such as validated measures, and unified recruitment strategies, such as partnering with clinic staff and attending community events.

The principle of collaboration is rooted in the idea that stakeholders provide expert community knowledge. Research shows that people are more likely to engage positively with projects that reflect the interests of their communities [49]. Meaningful collaboration involves two-directional communication, partnership, and shared leadership between researchers and stakeholders [49]. Badger et al. included stakeholder participation at every stage of the research process and found that culturally appropriate, accessible interventions improve survivors' and caregivers' quality of life [24]. Stakeholder-researcher collaboration ensures that the research meets the community's needs.

#### 3.3.1 Collaborating with others

Collaboration was vital in developing culturally tailored study materials. At start-up, we formed a focus group to ensure materials were culturally and linguistically appropriate. Bilingual CHWs, local health ambassadors, previous participants, and a University's Equity, Diversity, and Inclusion Office member reviewed intervention materials in English and Spanish. Their feedback was instrumental in revising materials for cultural relevance. For instance, they recommended renaming a printed intervention piece from “toolkit” to “handbook,” finding “toolkit” too clinically positioned and off-putting. They also helped select appropriate images to illustrate a symptom and strategy for the Handbook [52]. A CAB encouraged the team to include past participants' quotes in brochures to highlight the trial's potential benefits for survivors and caregivers.

All interventions, instruments, and materials were offered in English and Spanish using an adapted version of Brislin's translation model for cross-cultural research. Brislin's model ensures cultural and functional equivalence by translating and back-translating quantitative instruments between languages [53]. Initially, a bilingual expert translates the material from the source language (English) into the target language (Spanish), and a second bilingual expert back-translates it to the source language. Discrepancies between the back-translation and the original translation are assessed, retranslated, and back-translated again [53]. The process is repeated until the source document and target language agree. We used this method for standardized instruments like the Spanish language PROMIS© (Patient-Reported Outcomes Measurement Information System) scales [54], with adjustments based on focus group feedback. The group found that some retranslations were necessary to convert universalist translation to regional translation [55]. A certified translator signed off all translated documents to ensure linguistic accuracy.

To streamline recruitment, the team created a bilingual webpage shared by active studies and approved by the Institutional Review Board (IRB). Promotional materials included the site's URL or QR code. A secure, HIPAA-compliant survey within the Research Electronic Data Capture (REDCap) platform was linked to the site, inviting potential participants to submit their contact details. A trained team member promptly contacted those interested and triaged them to the most appropriate study based on their needs. Additionally, we used REDCap to log past and upcoming events and track recruiter progress. This collaborative approach reduced the potential burden on community stakeholders and minimized competing priorities among studies.

### 3.4 Community Engagement

The social-ecological model views the community as influential in clinical trial recruitment and retention, and researchers should consider engaging with diverse populations as critical [56]. Researchers who interact at the community level can better address concerns, instill trust, and challenge more significant health equity issues. Cultural brokers, who have ties to target populations, can help researchers design meaningful research and approach otherwise hard-to-reach groups [57]. In a health care setting, they are linguistically and culturally diverse community members who bridge culture, language, and values to improve patient outcomes [58]. Cultural brokers can connect researchers with diverse survivors and caregivers by initiating connections with community insiders. For example, developing meaningful relationships with individuals from CHW organizations, such as promotoras, can foster community engagement in research with a shared health equity mission. To meet diverse recruitment goals, research teams should prioritize relationships with FQHCs and Rural Health Clinics, which provide health care to populations in medically disadvantaged areas. Additionally, researchers in regions with foreign consular offices can connect with diverse populations through consulate social and civil services, offering further opportunities for engagement.

First, recognizing the community as a unit of identity [59], the team respected community values and used a networking approach to develop fruitful relationships with individuals from organizations in our region, including border community health centers and FQHCs. Cultural insiders on the team were vital in establishing trust and acceptance, acting as brokers between the investigators and community stakeholders. Maintaining a visible presence in the community fostered collective engagement and provided opportunities to reach diverse members.

Recruiters were innovative with community outreach. In addition to health fairs and cancer events, the team presented at Veterans of Foreign Wars (VFW) posts, local Lions International chapters, the Mexican Consulate, and survivorship groups. The most successful events were those enriched in culture with regional food, music (e.g., mariachi), and dancing (e.g., folklorico). When invited, team members engaged in conversations about cancer survivorship and caregiving, answered questions about the research, and participated in cultural celebrations with the community.

The research team maintained a dedicated presence at the academic cancer center in our catchment area. Staff built relationships with the cancer center's social workers, nurse navigators, and clinical oncology teams through respectful communication and awareness of their time. Recruiters created concise, visual summary guides for providers to reference each study's criteria. With IRB approval, pre-screening medical records allowed the team to approach only those who were eligible and permitted by the oncologist. By respecting the clinical staff's time and the physician-patient relationship, the team secured an opportunity to maintain an informational table in the cancer center lobby.

The team's lead recruiter served as a cultural broker. As a veteran community health worker (CHW), she deeply understood the local and regional community in our catchment area. This knowledge helped bridge cultural gaps between academic researchers and the community. Lastly, survivors and caregivers who found the study beneficial expressed satisfaction within their social networks, and many offered to help ‘get the word out’ in the community. In turn, survivors and caregivers in diverse and underserved communities heard about the study and contacted us to learn more about eligibility.

### 3.5 Partnership

When applying the six recruitment guiding principles, it is critical to consider key informants' and cultural insiders' roles. We expand on this idea using informal advisors as partners. Informal advisors, or those with a less formal role in research, can be a valuable source of information. Informal advisors include past participants or community members with deep cultural knowledge or shared experience to help guide the researcher. Although they may not serve on a CAB, informal advisors can support the research team significantly, facilitating co-learning between the community and researchers. By listening to informal advisors, researchers can better understand and address the community's needs in their projects.

#### 3.5.1 Partnering on the research

Past participants served as informal advisors; some assumed a more formal role. In some cases, former participants contacted the research team after completing the study to ask how they could help volunteer their time. As a result, volunteers completed IRB training because bilingual, Spanish-speaking volunteers were critical in helping engage diverse survivors and caregivers. These individuals connected us with local promotoras, CHWs, and community stakeholders.

Community members, informal advisors, and cultural insiders informed the researchers of deficiencies in cancer-supportive care. We listened and learned that Hispanic and rural survivors in our catchment area lacked services, and caregivers lacked support. This information highlighted the community's need and guided the team to prioritize the recruitment of underserved and diverse dyads.

### 3.6 Careful Matching

Careful matching involves systematically pairing like characteristics between the research team and the target population [42], helping researchers understand community perceptions at an individual level [57]. Linguistically matching research staff with participants can help remove barriers, and connecting through regional dialects and colloquialisms builds trust. Furthermore, understanding a diverse community's sociocultural distinctions and deliberately matching on shared experiences and values can reduce health disparities and improve recruitment and retention [42,51]. Senior researchers should consider the cultural competency framework to prioritize matching characteristics between the team and community members.

#### 3.6.1 Matching carefully to remove barriers

We prioritized language and culture as matching characteristics based on regionally specific SDOH. Staff who interacted with potential participants were bilingual, bicultural, and had personal connections to cancer as caregivers or survivors. Beyond cancer experience, staff who regularly interacted with participants in our catchment area shared nationality or place of origin.

## 4 Discussion/conclusion

This paper describes guidelines and strategies for recruiting and retaining diverse and underserved cancer survivors and caregiver dyads in clinical research, drawing on SHINE Cancer Research group exemplars. Six recruitment guiding principles of Team Development, Shared Passion, Collaboration, Community Engagement, Partnership, and Careful Matching have emerged as best practices for cancer clinical trials in our catchment area. Similar models focused on reducing barriers to clinical trials have successfully recruited diverse and underserved populations through a community-informed approach [51,60,61]. Strategies to recruit and retain diverse survivor-caregiver dyads in the exemplars were developed and operationalized from shared best practices among EERC and other successful frameworks [51,60]. Implementing the strategies outlined in this paper has resulted in recruitment rates of 40–100 % minority participants in trials [24,40,41,62]. Current and active trials designed to increase cancer health equity among diverse survivor-caregiver dyads utilize the six recruitment guiding principles [63,64].

Connecting with diverse and underserved populations for research requires deliberate self-reflection by the researcher and the team. Cultural competency should be central to team development, focusing on integrating cultural values, beliefs, and language into all aspects of the research and consciously assessing behaviors, attitudes, and biases. Research team members with a passion for cancer health equity, a commitment to community outreach, and shared experiences create a unified and authentic team that is relatable and approachable. These elements, coupled with carefully matching like-characteristics between researchers and participants, prioritize cultural competency throughout the research.

Effective community engagement requires internal collaboration and streamlining recruitment procedures may reduce community burden. Shared data platforms allow collaborating studies to triage potential participants into a study that best fits their needs. Stakeholder input for outward-facing materials is critical to successful collaboration. Finally, community engagement necessitates cultural insiders and informal advisors as partners in the research. Leveraging relationships with regionally focused health organizations and arranging innovative community outreach expands the footprint of the research. Trusted community partners can help connect with hard-to-reach populations, making the research process more inclusive and impactful.

In conclusion, underrepresentation in research limits population-specific data, risking ineffective clinical decisions and poorly informed health policies. Socioeconomic and sociocultural factors, such as low SES, mistrust, and language barriers, further impede health equity in research. We tailored strategies to engage rural, borderland, and predominantly Spanish-speaking communities in our unique region of seasonal residents and a significantly low SES population. As effectiveness may vary in different demographic contexts, future research could benefit by applying the strategies in diverse catchment areas to assess their broader applicability. Our experience shows the six recruitment guiding principles are an innovative and effective tool for research teams to recruit and retain diverse and underserved cancer survivors and caregiver dyads, thereby promoting health equity in regions with cancer disparities.

## CRediT authorship contribution statement

**Mary Hadeed:** Writing – original draft, Visualization, Conceptualization. **Terry A. Badger:** Writing – review & editing, Visualization, Supervision, Conceptualization. **Chris Segrin:** Writing – review & editing, Visualization, Conceptualization. **Rogelio Robles-Morales:** Writing – review & editing. **Samantha J. Werts-Pelter:** Writing – review & editing.

## Funding

This work was supported by the 10.13039/100000048American Cancer Society [grant number CHERC-MSI-21-167-01-CHERC-MSI]

## Declaration of competing interest

The authors declare that they have no known competing financial interests or personal relationships that could have appeared to influence the work reported in this paper.

## References

[1] Chen M.S., Lara P.N., Dang J.H.T., Paterniti D.A., Kelly K. (2014). Twenty years post-NIH Revitalization Act: enhancing minority participation in clinical trials (EMPaCT): laying the groundwork for improving minority clinical trial accrual. Cancer.

[2] Turner B.E., Steinberg J.R., Weeks B.T., Rodriguez F., Cullen M.R. (2022). Race/ethnicity reporting and representation in US clinical trials: a cohort study. Lancet Reg. Health Am..

[3] Camidge D.R., Park H., Smoyer K.E. (2021). Race and ethnicity representation in clinical trials: findings from a literature review of Phase I oncology trials. Future Oncol..

[4] Bebi T. (2022). ASCO Quality Care Symposium.

[5] Unger J.M., Cook E., Tai E., Bleyer A. (2016). The role of clinical trial participation in cancer research: barriers, evidence, and strategies. Am. Soc. Clin. Oncol. Educ. Book.

[6] Bharucha A.E., Wi C.I., Srinivasan S.G., Choi H., Wheeler P.H., Stavlund J.R., Keller D.A., Bailey K.R., Juhn Y.J. (2021 Jul 12). Participation of rural patients in clinical trials at a multisite academic medical center. J. Clin. Transl. Sci..

[7] Rural Health Information Hub (July 18, 2022). Cancer prevention and treatment in rural areas overview. Ruralhealthinfo.org. https://www.ruralhealthinfo.org/topics/cancer#:%7E:text=While%20there%20are%20comparable%20cancer.

[8] Bhatia S., Landier W., Paskett E.D. (2022). Rural–urban disparities in cancer outcomes: opportunities for future research. J. Natl. Cancer Inst..

[9] Sekar R.R., Maganty A., Stensland K.D., Herrel L.A. (2024 Aug). Association of community-level social vulnerability with clinical trial discussion and participation among cancer survivors. JCO Oncol. Pract..

[10] National Cancer Institute (April 1, 2022). Rural urban disparities in cancer. gis.cancer.gov. https://gis.cancer.gov/mapstory/rural-urban/index.html.

[11] Unger J.M., Gralow J.R., Albain K.S., Ramsey S.D., Hershman D.L. (2016). Patient income level and cancer clinical trial participation. JAMA Oncol..

[12] National Cancer Institute (July 18, 2024). Rural cancer control | division of cancer control and population sciences (DCCPS). cancercontrol.cancer.gov.

[13] Kennedy A.E., Vanderpool R.C., Croyle R.T., Srinivasan S. (2018). An overview of the national cancer institute's initiatives to accelerate rural cancer control research. Cancer Epidemiol. Biomarkers Prev..

[14] Marie Davidson Tara, Le H., Campbell E. (2023). Clinical trial deserts: US urban vs rural patient enrollment among patients with advanced cancer in phase 1 clinical trials at a major cancer center. J. Clin. Oncol..

[15] Oyer R.A., Hurley P., Boehmer L. (2022). Increasing racial and ethnic diversity in cancer clinical trials: an American Society of Clinical Oncology and Association of Community Cancer Centers joint research statement. J. Clin. Oncol..

[16] Beltrán Ponce SE., Thomas C.R., Diaz D.A. (2022). Social determinants of health, workforce diversity, and financial toxicity: a review of disparities in cancer care. Curr. Probl. Cancer.

[17] Arevalo M., Heredia N.I., Krasny S. (2016). Mexican-American perspectives on participation in clinical trials: a qualitative study. Contemp. Clin. Trials. Commun..

[18] Sabo S., Shaw S., Ingram M. (2014). Everyday violence, structural racism and mistreatment at the US–Mexico border. Soc. Sci. Med..

[19] Carvajal S.C., Rosales C., Rubio-Goldsmith R. (2012). The border community and immigration stress scale: a preliminary examination of a community responsive measure in two southwest samples. J. Immigr. Minority Health.

[20] Aarp for A. (May 14, 2020). https://www.aarp.org/pri/topics/ltss/family-caregiving/caregiving-in-the-united-states/.

[21] Kent E.E., Mollica M.A., Buckenmaier S., Wilder Smith A. (2019 Aug). The characteristics of informal cancer caregivers in the United States. Semin. Oncol. Nurs..

[22] Holmstrom A.J., Wyatt G.K., Sikorskii A., Musatics C., Stolz E., Havener N. (2016). Dyadic recruitment in complementary therapy studies: experience from a clinical trial of caregiver-delivered reflexology. Appl. Nurs. Res..

[23] Badger T.A., Sikorskii A., Segrin C. (2019). Contextual and cultural influences on caregivers of Hispanic cancer survivors. Semin. Oncol. Nurs..

[24] Badger T.A., Segrin C., Hepworth J.T., Pasvogel A., Weihs K., Lopez A.M. (2012). Telephone-delivered health education and interpersonal counseling improve quality of life for Latinas with breast cancer and their supportive partners. Psycho Oncol..

[25] Kershaw T., Ellis K.R., Yoon H., Schafenacker A., Katapodi M., Northouse L. (2015). The interdependence of advanced cancer patients' and their family caregivers' mental health, physical health, and self-efficacy over time. Ann. Behav. Med..

[26] Litzelman K., Berghoff A., Stevens J., Kwekkeboom K. (2023). Predictors of psychoneurological symptoms in cancer caregivers over time: role of caregiving burden, stress, and patient symptoms. Support. Care Cancer.

[27] Cao Q., Gong J., Chen M., Lin Y., Li Q. (2022). The dyadic effects of self-efficacy on quality of life in advanced cancer patient and family caregiver dyads: the mediating role of benefit finding, anxiety, and depression. J Oncol.

[28] Thomas Hebdon M.C., Badger T.A., Segrin C., Crane T.E., Reed P. (2022). Social and cultural factors, self-efficacy, and health in Latino cancer caregivers. Cancer Nurs..

[29] Langer S.L., Brown J.D., Syrjala K.L. (2009). Intrapersonal and interpersonal consequences of protective buffering among cancer patients and caregivers. Cancer.

[30] Allen M.P., Johnson R.E., McClave E.Z., Alvarado-Little W. (2020). Language, interpretation, and translation a clarification and reference checklist in service of health literacy and cultural respect. NAM Perspect.

[31] Lopez C., Vazquez M., McCormick A.S., Familismo (2022). Respeto, and bien educado: traditional/cultural models and values in Latinos. Family Literacy Practices in Asian and Latinx Families.

[32] Bisht J., Rawat P., Sehar U., Reddy P.H. (2023). Caregivers with cancer patients: focus on Hispanics. Cancers.

[33] Pinquart M., Sörensen S. (2005). Ethnic differences in stressors, resources, and psychological outcomes of family caregiving: a meta-analysis. Gerontol..

[34] Knight B.G., Robinson G.S., Flynn Longmire C.V., Chun M., Nakao K., Kim J.H. (2002). Cross cultural issues in caregiving for persons with dementia: do familism values reduce burden and distress?. Ageing Int..

[35] Skaff M.M., Pearlin L.I. (1992). Caregiving: role engulfment and the loss of self. Gerontol..

[36] Perrin P.B., Panyavin I., Morlett Paredes A. (2015). A disproportionate burden of care: gender differences in mental health, health-related quality of life, and social support in Mexican multiple sclerosis caregivers. Behav. Neurol..

[37] Fenton A., Ornstein K., Dilworth-Anderson Peggye (2022). Racial and ethnic disparities in cancer caregiver burden and potential sociocultural mediators. Support. Care Cancer.

[38] Sadigh G., Switchenko J., Weaver K.E. (2021). Correlates of financial toxicity in adult cancer patients and their informal caregivers. Support. Care Cancer.

[39] Sikorskii A., Badger T., Segrin C. (2023). A sequential multiple assignment randomized trial of symptom management after chemotherapy. J. Pain Symptom Manag..

[40] Badger T., Segrin C., Crane T.E. (2024). A sequential multiple assignment randomized trial of symptom management for cancer survivors during treatment and their informal caregivers. Support. Care Cancer.

[41] Badger T.A., Segrin C., Sikorskii A. (2019). Randomized controlled trial of supportive care interventions to manage psychological distress and symptoms in Latinas with breast cancer and their informal caregivers. Psychol. Health.

[42] Matsuda Y., Brooks J.L., Beeber L.S. (2016). Guidelines for research recruitment of underserved populations (EERC). Appl. Nurs. Res..

[43] Beeber L.S., Meltzer-Brody S., Mandel M., Wheeler A., Hubbard G., Mills-Koonce R., Alvarez S. (2014, September). Council for the Advancement of Nursing Science: 2014 State of the Science Conference.

[44] Cartagena D., Matsuda Y., McGrath J.M. (2020). Recruitment of immigrant hispanic mothers in research: an application of the EERC guidelines. J. Transcult. Nurs..

[45] Hesse-Biber S. (2016). Doing interdisciplinary mixed methods health care research: working the boundaries, tensions, and synergistic potential of team-based research. Qual. Health Res..

[46] Mclaughlin S. (2024). When I say … positionality. Med. Educ..

[47] Wallington S.F., Dash C., Sheppard V.B. (2016). Enrolling minority and underserved populations in cancer clinical research. Am. J. Prev. Med..

[48] ALTA Language Services (July 29, 2024). Testing languages offered. ALTA Language Services. https://altalang.com/languages-offered/.

[49] Riccardi M.T., Pettinicchio V., Di Pumpo M. (2023). Community-based participatory research to engage disadvantaged communities: levels of engagement reached and how to increase it. A systematic review. Health Pol..

[50] (2023). Roots 2023 | azCHOW. AzCHOW. https://www.azchow.org/roots2023.

[51] Cunningham-Erves J., Joosten Y., Kusnoor S.V. (2023). A community-informed recruitment plan template to increase recruitment of racial and ethnic groups historically excluded and underrepresented in clinical research. Contemp. Clin. Trials.

[52] Badger T., Sikorskii A., Segrin C., Crane T., Hadeed M. (2023).

[53] Jones P.S., Lee J.W., Phillips L.R., Zhang X.E., Jaceldo K.B. (2001). An adaptation of Brislin's Translation Model for cross-cultural research. Nurs. Res..

[54] (March 27, 2023). Healthmeasures. PROMIS. http://www.healthmeasures.net.

[55] Badger T.A., Heitkemper M., Lee K.A., Bruner D.W. (2014). An experience with the patient-reported outcomes measurement information System: pros and cons and unanswered questions. Nurs. Outlook.

[56] Salihu H.M., Wilson R.E., King L.M., Marty P.J., Whiteman V.E. (2015). Socio-ecological model as a framework for overcoming barriers and challenges in randomized control trials in minority and underserved communities. Int J MCH AIDS.

[57] Rojo M., Jing J., Wells C., Rodriguez J., Prince L. (2024). Hispanics' perceptions of participation in research studies and solutions for improvement in participation. J Fam Med Community Health.

[58] Luig T., Naadu Ofosu N., Chiu Y.E. (2023). Role of cultural brokering in advancing holistic primary care for diabetes and obesity: a participatory qualitative study. BMJ Open.

[59] Israel B.A., Schulz A.J., Parker E.A., Becker A.B. (1998). Review of community-based research: assessing partnership approaches to improve public health. Annu. Rev. Publ. Health.

[60] Treweek S., Banister K., Bower P. (2021). Developing the INCLUDE Ethnicity Framework—a tool to help trialists design trials that better reflect the communities they serve. Trials.

[61] Chhatre S., Jefferson A., Cook R. (2018). Patient-centered recruitment and retention for a randomized controlled study. Trials.

[62] Badger T., Segrin C., Crane T.E. (2024). Symptom management interventions influence unscheduled health services use among cancer survivors and caregivers. J Cancer Surviv.

[63] Segrin C. (June 21, 2024). Need-based symptom management for rural-urban cancer survivors and caregivers. ClinicalTrials.gov identifier: NCT05360498. Updated. NCT05360498.

[64] Bea Jennifer (August 7, 2024). Symptom management lifestyle intervention with Hispanic cancer survivors and caregivers. ClinicalTrials.gov identifier: NCT05364372. Updated. NCT05364372.

